# A Bioinformatics Approach to Explore MicroRNAs as Tools to Bridge Pathways Between Plants and Animals. Is DNA Damage Response (DDR) a Potential Target Process?

**DOI:** 10.3389/fpls.2019.01535

**Published:** 2019-11-26

**Authors:** Massimo Bellato, Davide De Marchi, Carla Gualtieri, Elisabetta Sauta, Paolo Magni, Anca Macovei, Lorenzo Pasotti

**Affiliations:** ^1^Laboratory of Bioinformatics, Mathematical Modelling, and Synthetic Biology, Department of Electrical, Computer and Biomedical Engineering—Centre for Health Technology, University of Pavia, Pavia, Italy; ^2^Plant Biotechnology Laboratory, Department of Biology and Biotechnology “L. Spallanzani”, University of Pavia, Pavia, Italy

**Keywords:** bioinformatics, DNA damage response, microRNA, networks, *trans*-kingdom

## Abstract

MicroRNAs, highly-conserved small RNAs, act as key regulators of many biological functions in both plants and animals by post-transcriptionally regulating gene expression through interactions with their target mRNAs. The microRNA research is a dynamic field, in which new and unconventional aspects are emerging alongside well-established roles in development and stress adaptation. A recent hypothesis states that miRNAs can be transferred from one species to another and potentially target genes across distant species. Here, we propose to look into the *trans*-kingdom potential of miRNAs as a tool to bridge conserved pathways between plant and human cells. To this aim, a novel multi-faceted bioinformatic analysis pipeline was developed, enabling the investigation of common biological processes and genes targeted in plant and human transcriptome by a set of publicly available *Medicago truncatula* miRNAs. Multiple datasets, including miRNA, gene, transcript and protein sequences, expression profiles and genetic interactions, were used. Three different strategies were employed, namely a network-based pipeline, an alignment-based pipeline, and a *M. truncatula* network reconstruction approach, to study functional modules and to evaluate gene/protein similarities among miRNA targets. The results were compared in order to find common features, e.g., microRNAs targeting similar processes. Biological processes like exocytosis and response to viruses were common denominators in the investigated species. Since the involvement of miRNAs in the regulation of DNA damage response (DDR)-associated pathways is barely explored, especially in the plant kingdom, a special attention is given to this aspect. Hereby, miRNAs predicted to target genes involved in DNA repair, recombination and replication, chromatin remodeling, cell cycle and cell death were identified in both plants and humans, paving the way for future interdisciplinary advancements.

## Introduction

The classical definition describes microRNAs (miRNAs) as small non-coding, single-stranded molecules that bind to mRNA by sequence complementarity and inhibit gene expression through posttranscriptional regulation (Bartel, 2004; Pasquinelli, 2012). By doing so, miRNAs are involved in many cellular and developmental processes, acting as master-regulators of gene expression. It is well-known that miRNAs are evolutionarily conserved in eukaryotes, although some differences exist between animals and plants, mainly related to their biogenesis and target recognition mechanism (see reviews by Millar and Waterhouse, 2005; Moran et al., 2017). In plants microRNAs are produced in nucleus and exported to cytoplasm, whereas in animals pri-microRNA and pre-microRNA are produced in the nucleus while the microRNA/microRNA* are produced in the cytoplasm. Both plant and animal miRNAs associate with the RISC complex, indispensable for miRNA activity, in the cytoplasm. In animals, pri-miRNAs are first cleaved by Drosha RNase III while in plants this is carried out by Dicer-like (DCL)1. Plant miRNAs have a 2′-O-methylation on the 3′-terminal nucleotide which is not present in animal miRNAs. Considering the target recognition mechanisms, in plants this is based on near-perfect or perfect sequence complementarity (leading mostly to mRNA decay), whereas in animals the sequence complementarity is imperfect, mostly based on the ‘seed rule’ (base pairing to the 5′ end of miRNAs, especially nucleotides 2–7) (Lewis et al., 2005).

Emerging research proposes a novel and controversial hypothesis indicating that miRNAs can be transferred from one species to another and potentially target genes across distant species. This concept has been developed starting from evidence showing that small RNAs can move from cell to cell (Molnar et al., 2010) and can act in gene silencing (RNA interference) across species (see reviews by Han and Luan, 2015; Weiberg et al., 2015). While the transfer of miRNAs from plants or humans/animals to their pathogens (Valadi et al., 2007; LaMonte et al., 2012; Buck et al., 2014) is less disputed, the situation gets more complicated when addressing the plant miRNA transfer to humans. This is due to several open questions and contrasting results regarding plant miRNA stability, abundance, mode of action, and validation of potential targets in human cells (Dickinson et al., 2013; Tosar et al., 2014; Micó et al., 2016; Cavallini et al., 2018). The first direct indication that ingested plant miRNAs, derived from food, can target genes in a cross-kingdom fashion had been provided by Zhang et al. (2012). The authors showed that a rice miRNA (osa-miR168a) stably exists in the sera and tissues of animals and humans and it specifically targets the liver low-density lipoprotein (LDL) receptor adapter protein 1 (LDLRAP1), decreasing the removal of LDL from plasma. Briefly, this research proposes that plant miRNAs are released from destroyed cells (during mechanical mastication) and transferred to the intestinal epithelial cells, where they could be incorporated into vesicles (exosomes or microvesicles) and enter the circulatory system to be delivered to targeted cells. Plant miRNAs can resist the activity of digestive enzymes and low pH throughout the gastrointestinal tract due to their methylation and high GC content (Zhang et al., 2012; Philip et al., 2015; Zhou et al., 2015). Moreover, immunoprecipitation experiments with anti-AGO2 antibodies have shown that miR168a associates with AGO2 in Caco-2 cells, thus enabling miRNAs’ function (Zhang et al., 2012). This was also confirmed in another study where immunoprecipitation data revealed that honeysuckle (*Lonicera japonica*) miR2911 associated with the AGO2 complex in microvesicles (Zhou et al., 2015). In this study, miR2911 has been demonstrated to be resistant to processing and proposed to target genes involved in the resistance to viral influenza. Hence, resistant exogenous plant miRNAs may regulate multiple target genes based on sequence complementarity, similarly to how endogenous miRNAs act (Liu et al., 2017). This concept expands the known types of miRNA functions to key natural bioactive compounds with potential health promoting benefits (depending on the mRNA target). So far, compelling evidence has demonstrated that plant miRNAs are present in human/animal plasma and these miRNAs usually belong to evolutionary conserved families (Vaucheret and Chupeau, 2012; Zhang et al., 2012; Liang et al., 2014; Yang et al., 2015a; Yang et al., 2015b; Cavalieri et al., 2016). Plant miRNAs not only from edible plant species (rice, cabbage, broccoli, watermelon, soybean, strawberry, olive) but also from model (Arabidopsis, poplar) and medicinal plants (Moringa, honeysuckle, turmeric, ginger) had been evaluated for their potential *trans*-kingdom transfer (Zhang et al., 2012; Liang et al., 2014; Zhou et al., 2015; Cavalieri et al., 2016; Chin et al., 2016; Pirrò et al., 2016; Liu et al., 2017; Sharma et al., 2017; Minutolo et al., 2018).

Aside from the biomedical interest, miRNAs *trans*-kingdom interactions can be useful to better understand evolutionary distant conserved pathways. Some examples of preserved pathways between plants and animals include the innate immune signaling pathways (Ausubel, 2005), programmed cell death (PCD)-related pathways (Godbole et al., 2003; Lord and Gunawardena, 2012), some basic functions (e.g. Ca^2+^ATPase, Ca^2+^/Na^+^-K^+^ ion exchanger) of calcium signaling pathway (Nagata et al., 2004), and the DNA damage response (DDR) (Yoshiyama et al., 2013; Nikitaki et al., 2018). Among these, DDR is defined as a complex signal-transduction pathway consisting of DNA damage sensors, signal transducers, mediators, and effectors which in turn activate a series of events (e.g. phosphorylation cascades) that lead to the regulation of downstream processes (e.g. cell cycle checkpoint, DNA repair), common between the plant and animal kingdoms (Yoshiyama et al., 2013). The involvement of miRNAs in the regulation of DDR players is quite recent and insufficiently explored, especially within the plant kingdom. Conversely, studies in human cells have already shown that miRNAs are involved in the regulation of DDR-associated genes and their activity is intricately weaved with traditional elements such as ATM (ataxia-telangiectasia mutated) and p53 (Kato et al., 2009; Landau and Slack, 2011; Wan et al., 2011). In plants, some miRNAs (e.g. osa-miR414, osa-miR164e, and osa-miR408), have been demonstrated to target specific helicases with roles in DNA repair, recombination, replication and translation initiation (Macovei and Tuteja, 2012; Macovei and Tuteja, 2013).

The current work aims to investigate the *in silico trans*-kingdom valence of plant miRNAs as a potential tool to bridge conserved pathways between plant and human cells, inquiring their implication in DDR. To do so, a multi-faceted bioinformatics approach was developed by combining and evaluating different data- or knowledge-driven resources and tools. The model legume *Medicago truncatula* (barrel medic) has been chosen as target for this analysis because of its potential medicinal properties (high content in saponins) (Tava et al., 2011), sequenced genome and availability of different databases (Goodstein et al., 2012), as well as its conserved synteny among legumes (Gujaria-Verma et al., 2014; Lee et al., 2017) which can offer the possibility of translational applications to other economically relevant species. Moreover, in view of promoting future sustainable agriculture practices and food security, microgreens, defined as seedlings harvested when the first leaves appear, are gaining momentum as novel functional food sources with high nutritional content and health-promoting benefits (Choe et al., 2018). In this context, legume species previously used only as fodder, like *Trifolium* spp., *Medicago* spp. and *Astragalus* spp., are now being proposed as microgreens for human consumption since they had been demonstrated to contain high protein and phytochemical contents as well as low levels of carbohydrates (Butkutė et al., 2018). Hence, starting from a collection of *M. truncatula* miRNAs, we retrieved candidate targets in plant and human transcriptomic datasets and analyzed them with different strategies: *(1)* a gene network-based strategy was used to compare the targeted biological processes in plant and human, using an *Arabidopsis thaliana* homology-based approach for plant network reconstruction; *(2)* an alignment-based strategy was used to identify nucleotide and protein similarities between *M. truncatula* and *Homo sapiens* putative targets; *(3)* another network-based strategy was carried out by using a *de novo* reconstructed *M. truncatula* gene network to further assess the common biological processes targeted in human and barrel medic. All the above-mentioned strategies have been used for the common purpose of identifying shared features (e.g. microRNAs targeting similar processes) between these distantly related organisms.

## Materials and Methods

**Figure 1:**
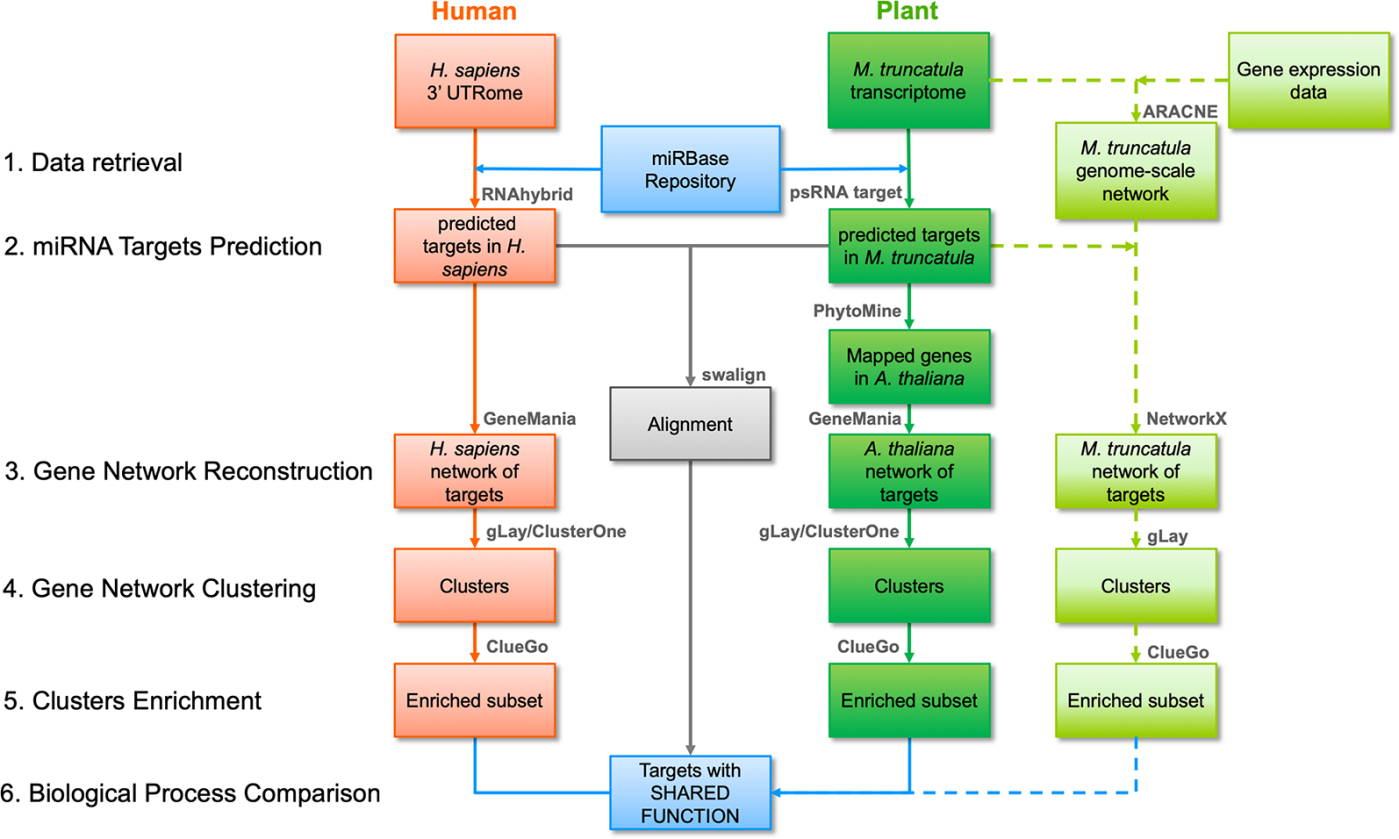
Bioinformatic workflow followed in this work including network- and alignment-based analysis pipelines. The main steps of the network-based pipeline are numbered on the left, at the same level as the pipeline blocks indicating input and output of each step. Red and dark green blocks indicate human and plant inputs/outputs, respectively, and data flow is reported with arrows. The main software tools or functions (detailed in the main text) are summarized above each block. Light green blocks indicate inputs/outputs for the *Medicago truncatula* network-based pipeline, also including genome-scale network construction, and its data flow is reported as dashed arrows. The outputs of the alignment-based pipeline are reported as a single grey block indicating the sequences with significant similarity after alignment. Blue blocks indicate the initial and final data for human and plant in both analysis pipelines.

The workflow followed in this study is illustrated in **Figure 1** and its parts are discussed below. Three different strategies were employed, namely a network-based pipeline, an alignment-based pipeline, and a *M. truncatula* network reconstruction approach.

### Datasets

The list of *M. truncatula* miRNAs was retrieved from miRBase (Kozomara et al., 2019) and included 756 sequences, among which 426 were unique. The human 3′ UTRome sequence dataset was retrieved from the psRNATarget tool web site (Dai et al., 2018) and included 21,233 sequences, among which 18,167 were relative to unique genes. The *M. truncatula* transcript dataset (Mt4.0 v1) was retrieved from the psRNATarget tool web site and included 62,319 transcripts, corresponding to 50,894 unique genes.

The gene sequences and the related protein sequences of the predicted targets were retrieved from the NCBI RefSeq database (for human targets) (O’Leary et al., 2016), and from the annotated coding sequence and protein datasets from the *M. truncatula* Genome Database (for plant) (Krishnakumar et al., 2014).

Six microarray datasets from the ArrayExpress (Kolesnikov et al., 2015) repository were used: E-MEXP-1097 (Benedito et al., 2008), E-MEXP-3719 (Verdier et al., 2013), E-MEXP-2883 (Tang, 2014), E-MEXP-3190 (Uppalapati et al., 2012), E-MEXP-3909 (Wang et al., 2016), and E-GEOD-43354 (Limpens et al., 2014). These amounted to a total of 117 raw expression samples (in CEL format) that were used for *M. truncatula* co-expression network reconstruction. The dataset samples measured under perturbed conditions (e.g. salt or drought stress, infections) were excluded. All the considered experiments were conducted on the same microarray platform (Affymetrix GeneChip Medicago Genome array), thereby avoiding genome annotation biases.

### miRNA Target Prediction

The psRNATarget (Dai et al., 2018) and RNAhybrid (Kruger and Rehmsmeier, 2006) online tools, specific for miRNA target prediction in plants and mammalians, respectively, were used. The list of *M. truncatula* unique miRNAs was used as input for both tools, together with the *M. truncatula* transcript dataset or the human 3′ UTRome (unless differently indicated). The 3′ UTR region was chosen under the assumption that plant miRNAs can regulate human targets in the same manner as endogenous human miRNAs (Bartel, 2004). This assumption is consistent with a number of recent bioinformatics works, which were in some cases further validated, leading to experimental evidence of cross-kingdom regulation (Shu et al., 2015; Chin et al., 2016; Zhang et al., 2016a; Hou et al., 2018; Zhao et al., 2018a). Despite this commonly performed assumption, it is worth noting that no *golden standard* exists for plant miRNA target prediction in a cross-kingdom context (Lukasik et al., 2018). A small number of other works additionally considered 5′ UTR and/or coding sequences as potential target regions (Liu et al., 2017; Lukasik et al., 2018; Mal et al., 2018). This was motivated by studies in which different transcript regions have been reported as non-3′ UTR targets for both endogenous and cross-kingdom regulations (Li et al., 2018; Wang et al., 2018). With the availability of additional validation studies and models for cross-kingdom regulation, this gap will be filled. Importantly, the proposed workflow can be easily adapted by changing the target sequences files.

The parameters of the two target prediction tools were set to obtain a balanced number of network nodes (about 700 for *A. thaliana* and *H. sapiens*) in the network-based pipeline, and of unique target transcripts (about 1,700 for *M. truncatula* and *H. sapiens*) in the alignment-based pipeline. A highly specific hybridization in seed region, typically occurring in plants, was set in psRNATarget, which was used to find plant target genes for network-based pipeline with the following parameters: number of top targets = 50, Expectation = 2.5, Penalty for G:U pair = 0.5, Penalty for other mismatches = 1, Extra weight in seed region = 1.5, Seed region = 2–13 nucleotides, Mismatches allowed in seed region = 0, HSP size = 19. The list of targets for the alignment-based pipeline was obtained *via* the same parameters as above except the number of top targets which was set to 15. The predicted target list from RNAhybrid was filtered by tuning the sole algorithm parameter that is Minimum Free Energy (MFE), whose threshold was set to −36.5 kcal/mol, while for the alignment-based pipeline it was −34.7 kcal/mol. In both cases, a maximum of 50 targets per miRNA was considered (Zhang et al., 2016a).

### Network-Based Pipeline

The lists of predicted targets were used to construct plant and human target networks using GeneMania, (Warde-Farley et al., 2010), and considering all the genetic and co-expression interactions available within the tool. Since GeneMania does not contain *M. truncatula* among the available organisms, the following procedure was used to construct a genetic interaction/co-expression network of *A. thaliana*, by mapping the homologous genes of the *M. truncatula* predicted targets list. The Phytomine tool (Goodstein et al., 2012) of the Phytozome portal (JGI) was used to obtain a mapping from the *M. truncatula* target genes to *A. thaliana* genes, based on homology. Correspondences between the species were considered with a relative threshold similarity above 85%.

**Figure 2:**
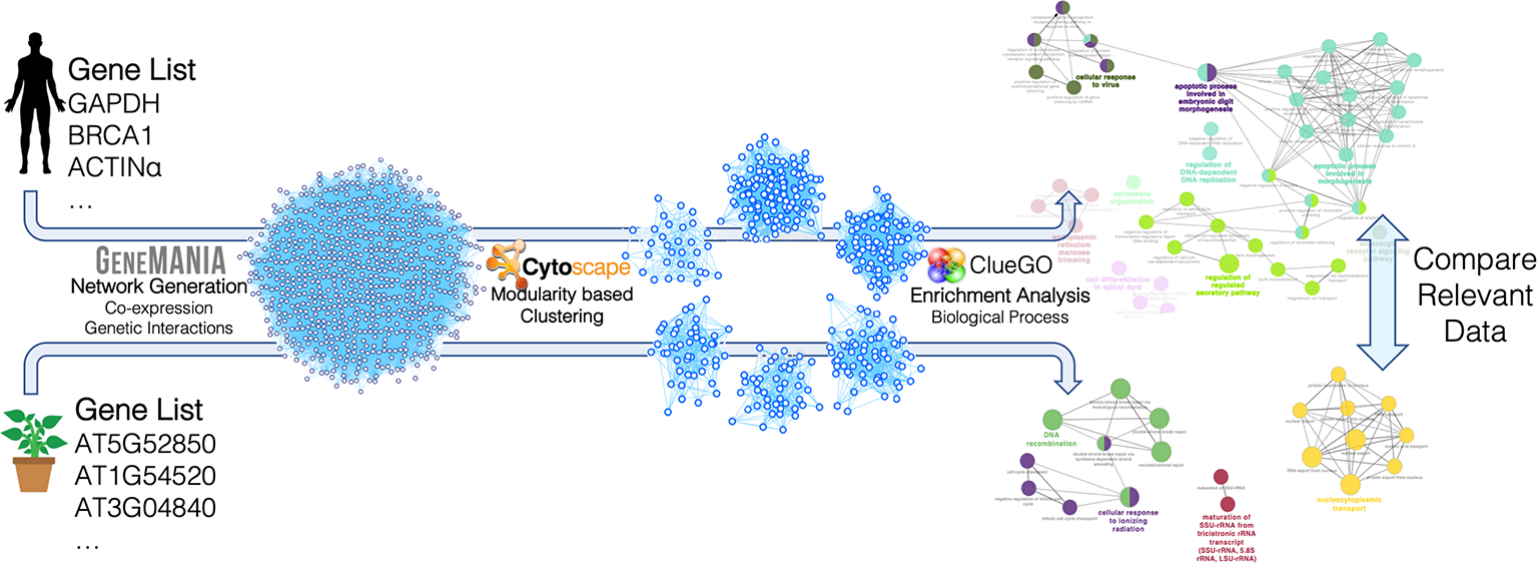
Construction and analysis steps of the miRNA target networks for *Arabidopsis thaliana* and *Homo sapiens*. The lists of miRNA target genes were used to construct genetic interaction/co-expression networks with GeneMania. The resulting networks were analyzed with Cytoscape and its applications. In particular, clustering was carried out with two different modularity-based methods (gLay and ClusterOne) and enrichment analysis was carried out with the ClueGO app to find enriched biological processes in each cluster. The resulting processes found for the two organisms were finally compared, taking into account the related target genes and miRNAs.

Human and plant networks were imported and analyzed using Cytoscape (v.3.7.1) (Shannon et al., 2003) and its applications. Clustering was carried out using the gLay (Su et al., 2010) and ClusterOne (Nepusz et al., 2012) algorithms, considering the networks as undirected and unweighted. ClusterOne was used with the following parameters: minimum size = 50, minimum density = 0.25, unweighted edges, node penalty = 2, haircut threshold = 0, merging method = Multi-pass, Jaccard similarity, overlap threshold = 0.15, seeding method from unused nodes. The gLay algorithm does not have free parameters. For each cluster, enrichment analysis was carried out using ClueGO (Bindea et al., 2009) to find statistically over-represented Gene Ontology (GO) terms in the Biological Process (BP) category, using a right-tail test with the Benjamini–Hochberg correction for multiple testing, and a 75% detail level. GO terms were considered for further analysis if they had *p*-value < 0.05 and if at least one of the related genes was present in the original target gene list (since GeneMania includes interactor genes not belonging to the input target list). The analysis procedure followed in this network-based pipeline is summarized in **Figure 2**.

### Alignment-Based Pipeline

**Figure 3:**
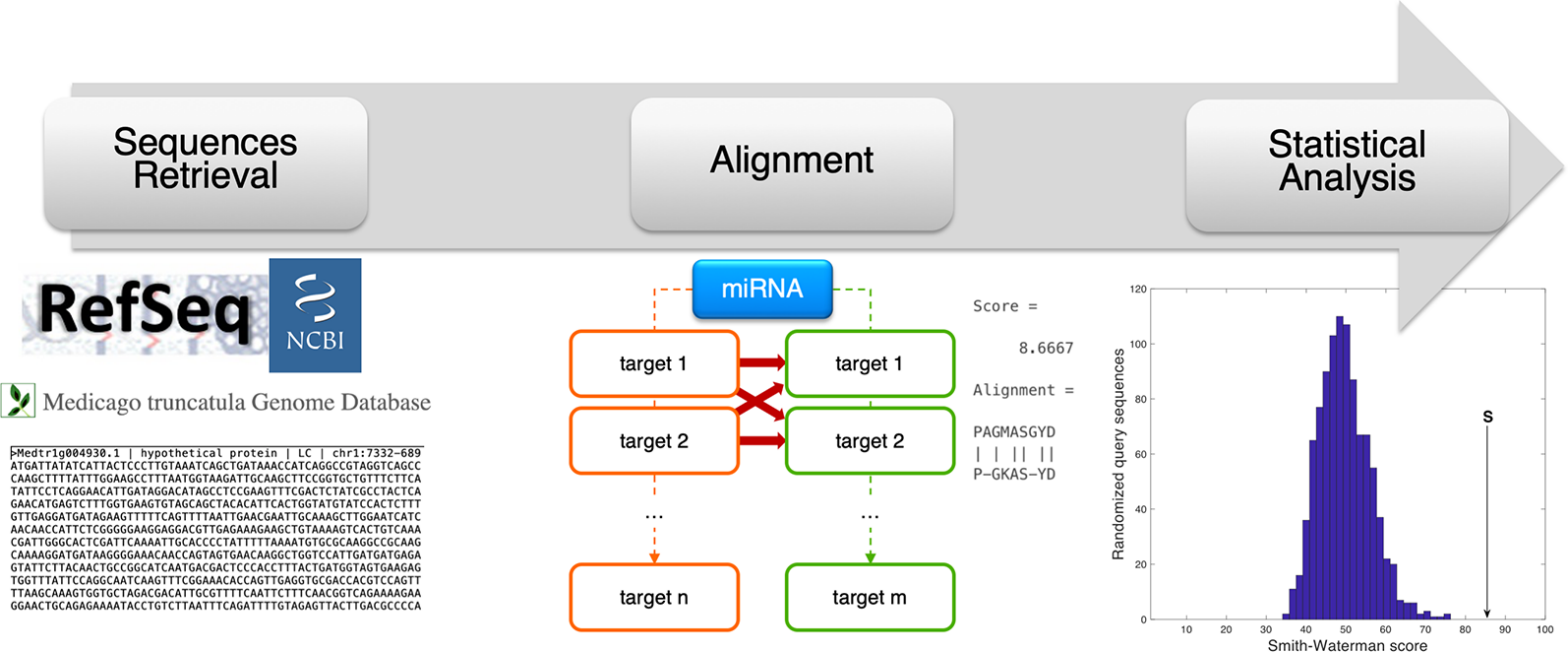
Schematic representation of the alignment-based pipeline. The coding sequence (CDS) and amino acid sequence corresponding to the miRNA target genes were retrieved from online resources (RefSeq and *Medicago truncatula* Genome Database). For each miRNA, the CDSs and amino acid sequences of human and plant targets were compared *via* sequence alignment (Smith-Waterman method, by the swalign Matlab function), to compute a similarity score (provided as swalign output) for each human-plant target pair. The statistical significance of the similarity score is finally computed following a randomization method in which, for every alignment, human sequences (CDS or protein) were randomized and the distribution of swalign scores was used to compute the *p*-value.

For each miRNA, the nucleotide coding sequence and protein sequence of the predicted transcript targets found in *M. truncatula* and *H. sapiens* were compared *via* sequence alignment. A custom MATLAB R2018a (MathWorks, Natick, MA, USA) script was programmed to automatically carry out this analysis and to evaluate the statistical significance of each comparison. The Smith–Waterman method (Smith and Waterman, 1981) was used to perform local alignment *via* the *swalign* function and get the optimal alignment score (in bits) as output. A random permutation-based statistical analysis was adopted to evaluate the significance of each alignment and to obtain a sequence length-independent scoring value (*p*-value) (Teng et al., 2014; Tiengo et al., 2015). Specifically, for each sequence comparison, 200 random permutations were constructed for the human nucleotide/protein sequence and an alignment was performed for each randomization. The resulting distribution of bits scores was used to obtain the final *p*-value as the number of alignments giving a bits score higher than the original one, divided by the number of randomizations. Low *p*-values correspond to statistically significant alignments with a considered threshold of 0.05. The analysis procedure followed in this alignment-based pipeline is summarized in **Figure 3**.

### Reconstruction of *M. truncatula* Co-Expression Network

Raw expression values were globally normalized using the Robust Multichip Average (RMA) method (Irizarry et al., 2003), and then annotated using the MedtrA17_4.0 *M. truncatula* reference genome assembly. Array probes mapping the same gene were median-averaged and those lacking functional annotation were discarded. Co-expression analysis of the obtained expression panel was performed *via* ARACNE (Margolin et al., 2006) by: *(i)* building the Mutual Information Matrix using the Spearman correlation, and then *(ii)* pruning the obtained interactions among all possible gene triplets with null mutual information. All the analyses were performed in the R environment, using the *limma* (Ritchie et al., 2015) and the *biomaRt* (Durinck et al., 2009) packages for the expression data preparation, and the *minet* package (Meyer et al., 2008) for the co-expression estimation with ARACNE. The obtained adjacency matrix was then used to reconstruct a co-expression network for the miRNA targets of *M. truncatula*, with a custom Python (v.2.7) script, exploiting the *NetworkX* package (Hagberg et al., 2008) to create networks in a Cytoscape-compatible format. The miRNA targets of *M. truncatula*, obtained as described above (see *miRNA Target Prediction* section), were mapped onto the network to extract their co-expression interactome. Target genes were mapped and all the co-expressed nodes which interact with at least one miRNA target node were included. The resulting sub-network was filtered, eliminating the smallest components, composed of single nodes or less than ten nodes because these are not informative in terms of interactions. The remaining giant component was considered as the final miRNA target gene network. The giant component was analyzed *via* the gLay clustering procedure and, as performed above for the other networks, the obtained clusters were subjected to the ClueGO enrichment step.

## Results

### Target Prediction

Following the *in silico* target prediction, a list of 3,468 *M. truncatula* transcripts (2,680 unique transcripts and 2,083 unique genes) was obtained. Conversely, 2,297 target transcripts of *M. truncatula*, corresponding to 1,739 unique transcripts and 1,376 unique genes, were considered for the alignment. Analogously, for the network-based analysis, a list of 936 target transcripts (825 unique transcripts and 758 unique genes) was obtained for *H. sapiens*. For the alignment-based pipeline, 2,226 target transcripts, which correspond to 1,754 unique transcripts and 1,549 unique genes, were obtained. The number of targets was tuned to obtain a balanced number of elements between the two species (see below). The target genes could be associated with one or more than one miRNA, as shown in **Supplementary Figure 1**.

### Mtr-miRNAs Targeting Shared Functions Between Plants and Humans From the Perspective of the Network-Based Approach

In this pipeline, we focused on the biological processes enriched among the genes targeted by the set of *M. truncatula* miRNAs to uncover shared functions between plant and human. Following the procedure summarized in **Figure 2**, plant and human miRNA target networks were constructed using the GeneMania web tool. While the construction of the human network was straightforward with this tool, the construction of the plant network relied on the mapping from *M. truncatula* targets to homologous genes in *A. thaliana*, thereby enabling to exploit the deep knowledge (datasets and resources) of *A. thaliana*, since *M. truncatula* is not currently supported by GeneMania. The resulting networks were analyzed using two different topology-based graph clustering methods, to decompose the target network, based on highly connected nodes, implying densely-interacting functional modules. The use of two different clustering methods was devised to increase the sensitivity of the pipeline for the detection of functional modules and, subsequently, associated biological processes. The features of the constructed target gene networks are summarized below:

1. *A. thaliana*—704 nodes (of which 20 were included by GeneMania as interactors), 13,752 edges, 4 gLay clusters, 6 ClusterOne clusters.
2. *H. sapiens*—753 nodes (of which 21 were included by GeneMania as interactors), 20,795 edges, 5 gLay clusters, 3 ClusterOne clusters.

The target genes and network clusters obtained in this analysis are reported for each species in the **Supplementary Dataset 1** file.

By performing an enrichment analysis for each cluster, we identified the common biological processes (GO terms) targeted by *M. truncatula* miRNAs in both species. The identified shared biological functions include ‘vesicle docking involved in exocytosis’ (GO:0006904), ‘modulation by virus of host morphology or physiology’ (GO:0019048), ‘cellular response to virus’ (GO:0098586), ‘positive regulation of posttranscriptional gene silencing’ (GO:0060148), and ‘branched-chain amino acid metabolic process’ (GO:0009081). The miRNAs and predicted target genes associated with the shared GO terms are listed in **Table 1**. Aside from the identical GO terminologies, other common processes were present in both networks (e.g. nucleic acid and amino acid metabolism, response to stress, signaling) (**Supplementary Dataset 1**).

**Table 1:** Common biological processes shared between *A. thaliana* and *H. sapiens* as resulted from the network-based approach. The ID corresponding to each GO term (GO ID) along with putatively target genes and corresponding miRNAs are provided.

Biological process	GO ID	*A. thaliana*		*H. sapiens*	
		Gene	miRNA	Gene	miRNA
Vesicle docking involved in exocytosis	GO:0006904	EXO70B1	mtr-miR5244	SNPH	mtr-miR399t-5p
		EXO70D1	mtr-miR2653a		
		EXO70H7	mtr-miR397-5p		
		KEU	mtr-miR5559-3p		
		SEC5A	mtr-miR7698-5p		
		SEC8	mtr-miR2679a		
Modulation by virus of host morphology or physiology	GO:0019048	AGO2	mtr-miR2673a	BCL2L11	mtr-miR5273
		DCP2	mtr-miR5238	KPNA4	mtr-miR169k
			mtr-miR2655b		
Cellular response to virus	GO:0098586	AGO1	mtr-miR168a	BCL2L11	mtr-miR5273
		SDE3	mtr-miR168c-5p	PUM2	mtr-miR160c
			mtr-miR2592a-3p	RIOK3	mtr-miR160a
			mtr-miR2592bm-3p		
Positive regulation of posttranscriptional gene silencing	GO:0060148	DRD1	mtr-miR2650	FXR1	mtr-miR482-3p
				PUM2	mtr-miR160c
Branched-chain amino acid metabolic process	GO:0009081	BCAT3	mtr-miR5212-3p	BCKDK	mtr-miR5273
		CSR1	mtr-miR2660	IVD	mtr-miR2640

Exocytosis generally implies the active (hence, energy-dependent) transport of newly synthesized lipids and proteins to the plasma membrane along with the secretion of vesicle-enclosed contents to the extracellular matrix. Experimental evidence that exocytosis-related events can be conserved between plants and animals has been recently provided by Zhang et al. (2016b), who demonstrated that specific molecules (namely endosidin 2) are able to inhibit EXO70 proteins, involved in intracellular vesicle trafficking, in both plants and animals. The fact that *M. truncatula* miRNAs are predicted to target functions related to exocytosis in both *A. thaliana* and *H. sapiens*, further indicate the conservation of these pathways between distant taxa. As an example, the KEU (KEULE) and SEC (EXOCYST COMPLEX COMPONENT) genes in Arabidopsis as well as the human SNPH (Syntaphilin) gene are part of the SNARE (soluble N-ethylmaleimide-sensitive factor attachment protein receptor) complex, which is required for vesicle docking and fusion (Lao et al., 2000; Karnik et al., 2015).

The hypothesis that innate immunity is an evolutionarily conserved process, started in the ancient unicellular eukaryote that pre-dated the divergence of the plant and animal kingdoms (Ausubel, 2005), may explain the shared plant and human response to virus. The network-based approach applied in our study allowed to find common players involved in the response to viral attacks in plants (AGO1, AGO2, DCP2, SDE3, DRD1) and humans (BCL2L11, KPNA4, PUM2, FXR1, RIOK3) (**Table 1**). Particularly, the KPNA4 (Karyopherin Subunit Alpha 4) mediates the nuclear import of human cytomegalovirus UL84 (Lischka et al., 2003), PUM2 (Pumilio RNA Binding Family Member 2) plays a role in cytoplasmic sensing of viral infection (Narita et al., 2014), and RIOK3 (right open reading frame-RIO Kinase 3) is involved in regulation of type I interferon (IFN)-dependent immune response, with a critical role in the innate immune response against DNA and RNA viruses (Feng et al., 2014).

The relationship between miR168a and AGO1 (ARGONAUTE) has been long studied and experimentally validated in plants (Vaucheret et al., 2006), whereas several other targets of the plant miR168a have been identified and/or validated in humans (Zhang et al., 2012; Javed et al., 2017). Aside being involved in miRNA biogenesis and regulation (Mallory and Vaucheret, 2010), AGO proteins have a myriad of other functions including plant antiviral responses and DNA repair (AGO2) (Harvey et al., 2011; Oliver et al., 2014; Carbonell and Carrington, 2015), miRNA-directed target cleavage (AGO5), and RNA-directed DNA methylation (AGO9) (Oliver et al., 2014). Differently, the validated osa-miR168a target in humans is LDLRAP1, with functions in cholesterol metabolism (Zhang et al., 2012), while other predicted targets included RPL34 (Large Ribosomal Subunit Protein EL34), ATXN1 (Ataxin-1), and ALS2 (Alsin Rho Guanine Nucleotide Exchange Factor) with roles in transcription, ribosome biogenesis, and cell trafficking (Javed et al., 2017). Other genes (ST8SIA1, RGS6, IL18RAP, PVR, SYN2, PPFIA1, ZDHHC18, B3GAT1) were predicted in our network-based pipeline to be targeted by mtr-miR168 in humans (**Supplementary Table 1**). This may be due to the fact that, even if miR168a is part of conserved miRNA family, some differences in nucleotide sequences are present among monocot and dicot species and these can alter the structural accessibility and target selection (Lang et al., 2019). When aligning the osa-miR168a with its counterpart in *M. truncatula*, the sequence similarity was of 80.95%, showing important mismatches in the seed region (**Supplementary Figure 2**). This, along with the fact that we took into consideration only the 3′ UTR region, explains the absence of LDLRAP1 among the mtr-miR168a targets in human. We confirmed this by comparing the *M. truncatula* (mtr-) or rice (osa-) miR168a targets found within the human transcript collection (retrieved from NCBI RefSeq) or in the 3′ UTRome, as performed in our pipeline. As expected, we found that no target was detected for both miRNAs in the 3′ UTRome, while targets in the LDLRAP1 coding sequence were found with relevant MFE. In particular, osa-miR168a showed a −35.3 kcal/mol MFE with LDLRAP1, which appeared in the top 5 of the miRNA targets, while mtr-miR168a showed a −33.8 kcal/mol MFE with LDLRAP1 in the 100^th^ position of the lowest-MFE target list (**Supplementary Figure 2**).

### miRNAs Targeting Shared Functions in *M. truncatula* and *H. sapiens* Through the Lens of the Alignment-Based Approach

Unlike the network-based pipeline in which over-represented biological processes were searched in the network of all the miRNA target genes, here we focus on sequence similarities among the targets of a given miRNA. In this approach, the analysis included alignments of every single targeted gene (and corresponding protein sequence) between *M. truncatula* and *H. sapiens*, resulting in a total number of 9,626 alignments (**Supplementary Dataset 2**). By applying a threshold *p*-value of 0.05 for nucleotide alignments, 2,735 sequences, corresponding to 115 miRNAs, resulted significant. These miRNAs were predicted to target a total of 315 genes in *M. truncatula* and 801 genes in *H. sapiens*, respectively. Similarly, when this threshold was applied for the protein alignments, from 697 alignments (including 81 miRNAs) 352 genes were identified in *H. sapiens* and 192 in *M. truncatula*. When considering both the gene and protein sequences, 242 similarities between plant and human transcripts were found, accounting for 93 genes (targeted by 54 miRNAs) in *M. truncatula* and 149 in *H. sapiens* (**Supplementary Dataset 2**).

Focusing on the identification of genes involved in similar functions between the two organisms, the main hits were related to transcription factors (including zinc finger proteins), hormone-responsive elements, and cell division (**Table 2**). The activity of transcription factors (TFs), consisting of the interaction with enhancers to coordinate gene expression, is a common denominator for all living forms. In eukaryotes, another level of regulation is given by miRNAs; these are known to target mostly TFs, at least in plants (Samad et al., 2017). Moreover, coordinated action of TFs and phytohormones guide most plant developmental processes as well as cellular proliferation and dedifferentiation (Long and Benfey, 2006). The predicted targets of miR164 belong to CUP and NAC families of TFs, and these had been previously validated in plants in other works (Fang et al., 2014). However, a piece of interesting information is the fact that this miRNA could target TFs also in human cells. For instance, ZXDC (predicted as a target of mtr-miR164b), belonging to the zinc finger X-linked duplicated (ZXD) family of TFs, is involved in the regulation of histocompatibility (Ramsey and Fontes, 2013). Elseway, HAND2 (Heart and Neural Crest Derivatives Expressed 2), putatively targeted by mtr-miR2673a, is a member of the helix-loop-helix family of TFs involved in cardiac morphogenesis, vascular development, and regulation of angiogenesis (McFadden et al., 2005). Another interesting fact is that this analysis predicted that conserved miRNA families (miR160, miR166) target genes with roles in hormone regulation in both *M. truncatula* (ABA response element-binding factor, auxin response factor) and *H. sapiens* (DYRK1B, HNF4A). In humans, DYRK1B (Dual Specificity Tyrosine Phosphorylation Regulated Kinase 1B), encoding for a nuclear-localized protein kinase, and HNF4A (Hepatocyte Nuclear Factor 4 Alpha), belonging to the nuclear hormone receptor family, are associated with steroid hormone activity (Sladek et al., 1990; Sitz et al., 2004). Other interesting hits revealed through this approach are presented in **Supplementary Table 2**. An example is represented by mtr-miR2600e, predicted to target an anthocyanin acyltransferase (Medtr2g089765) in *M. truncatula* and the UVSSA (UV Stimulated Scaffold Protein A) gene in *H. sapiens*. Anthocyanins are well-known secondary metabolites with antioxidant function, being able to mitigate photooxidative injury (e.g. UV irradiation) at the cellular and nuclear level by efficiently scavenging reactive oxygen species (Gould, 2004). UVSSA encodes a protein involved in ubiquitination and dephosphorylation of RNA polymerase II subunits, being involved in the transcription-coupled nucleotide excision repair (TC-NER) pathway associated with UV irradiation (Schwertman et al., 2013).

**Table 2:** Mtr-miRNAs and their putative target genes related to similar functions in *M. truncatula* and *H. sapiens* as revealed by the alignment-based approach. The genes and their respective accessions are provided for each organism.

mtr-miRNA	*M. truncatula*		*H. sapiens*	
	Accession	Gene	Accession	Gene
mtr-miR166d	Medtr2g086390	ABA response element-binding factor	NM_006484	DYRK1B
mtr-miR160a	Medtr5g061220	auxin response factor	NM_175914	HNF4A
mtr-miR2673a	Medtr2g014260	zinc finger C-x8-C-x5-C-x3-H type protein	NM_001170538	DZIP1L
mtr-miR2673a	Medtr4g082580	WRKY transcription factor 3	NM_021973	HAND2
			NM_032772	ZFN503
mtr-miR164b	Medtr2g078700	CUP-shaped cotyledon protein, putative	NM_001099694	ZNF578
	Medtr4g108760		NM_001040653	ZXDC
mtr-miR164d	Medtr3g435150	NAC transcription factor-like protein	NM_001018052	POLR3H
mtr-miR5287b	Medtr7g088980	cell division cycle protein-like/CDC48 protein	NM_001277742	CYP26B1

When comparing the network-based and alignment-based approaches, in the case of mtr-miR168a targets, it is possible to evidence the same predicted target in plants (AGO1) along with different predicted targets in humans (**Supplementary Table 2**, **Supplementary Dataset 2**). However, drawing the attention to the ‘response to virus’ function, it is possible to observe that this was hit with both approaches, as demonstrated by the common predicted target gene PVR (Poliovirus Receptor).

### Novel Co-Expression Network Reveals Shared Functions Targeted by mtr-miRNAs in Both *M. truncatula* and Humans

**Figure 4:**
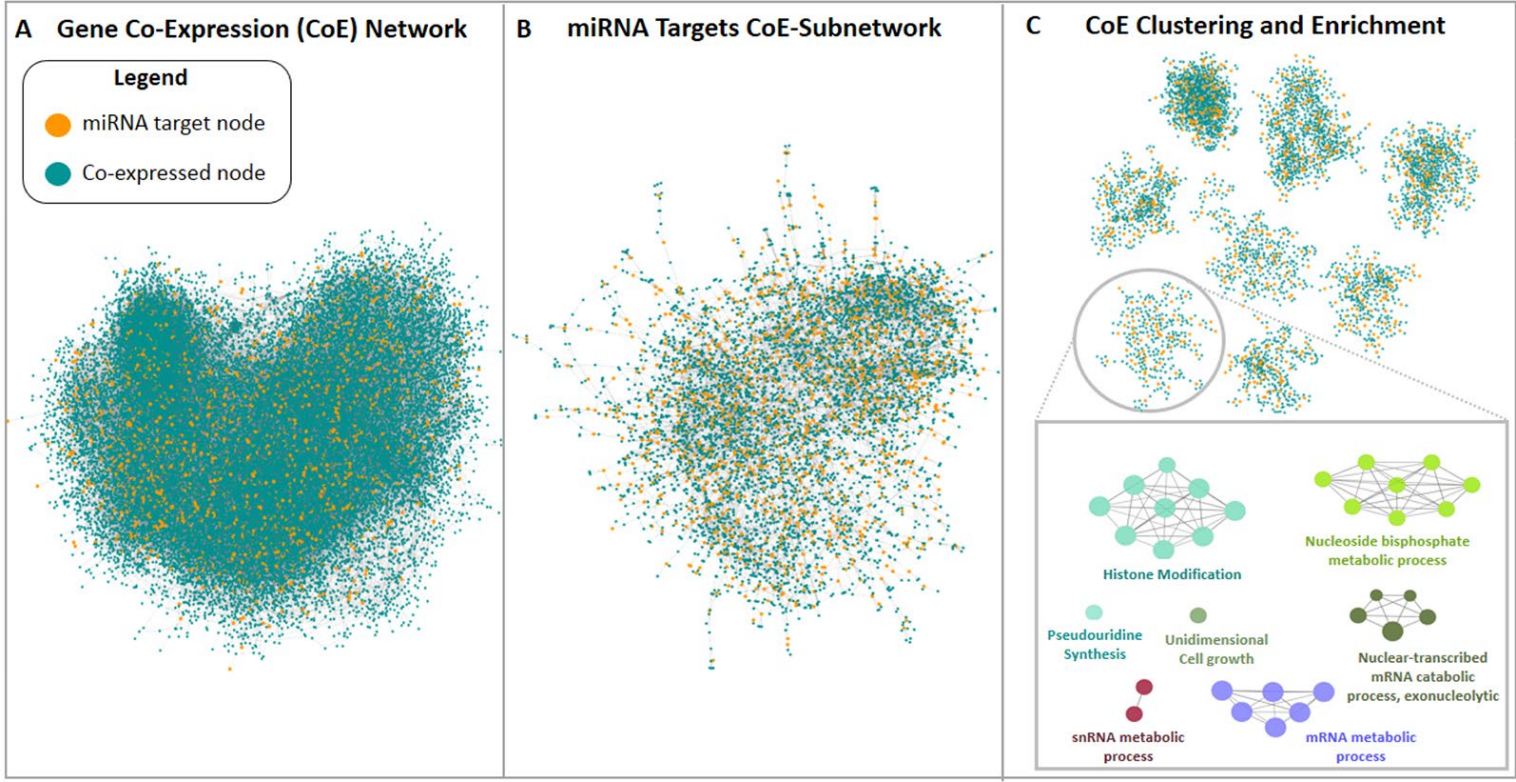
*Medicago truncatula* co-expression network construction and analysis pipeline. **(A)** Genome-scale co-expression network and **(B)** miRNA targets network are shown, where blue nodes represent genes not found among miRNA targets, while orange nodes are miRNA targets. **(C)** Representative set of the clusters resulted from the miRNA targets network analysis. Each cluster was analyzed *via* enrichment analysis using ClueGO.

The third approach used in this study pursued the construction of a new *M. truncatula* co-expression network using publicly available gene expression microarray datasets since this organism is not currently supported in readily usable bioinformatic tools for network analysis and construction. An expression panel of 24,777 genes was obtained and used to build a genome-scale co-expression network for *M. truncatula*. The resulting 24,777-node network had 62,857 undirected edges (**Figure 4A**). Among the 2,083 predicted target genes, 1,251 were mapped in this network, resulting in a sub-network of 6,081 nodes and 9,534 edges. The giant component of this sub-network included 5,943 nodes (of which 1,208 were target genes) and 9,405 edges (of which 3,102 were direct interactions among miRNA target nodes), as shown in **Figure 4B**. The clustering procedure found 45 clusters (**Figure 4C**) which were analysed *via* enrichment analysis. All the resulting GO terms along with the co-expressed genes and associated miRNAs are reported in the **Supplementary Dataset 3** file.

The herein generated network was compared with the first network-based approach made with the tools available for *A. thaliana* to evaluate if the two different network construction procedures lead to the same target biological processes, thereby assessing the robustness of the conclusions for the network-based approach. The GO terms identified within the *M. truncatula* network were mainly related to general processes such as metabolic pathways (e.g., nucleic acids, proteins, and carbohydrates metabolism), plant development (e.g., fruit, seed, embryo development), or hormone signaling (**Supplementary Dataset 3**). On the other hand, the Arabidopsis network was much more varied and specific (**Supplementary Dataset 1**), mostly because *A. thaliana* is *de facto* considered the plant model *par excellence* and hence, much more information, databases, and bioinformatics resources are available in this case. Despite a systematic comparison between the biological processes in the two plants could not be carried out, identical GO terms identified between *A. thaliana* and *M. truncatula* include exocytosis, folic acid metabolism, and thylakoid membrane organization (**Supplementary Table 3**). In this case, it can be underlined also the fact that some miRNAs (e.g., mtr-miR5559-3p, mtr-miR5558-3p, mtr-miR2662, mtr-miR5212-3p) are predicted to target the same genes/functions in both plant species. When comparing the shared biological processes between *M. truncatula* and *H. sapiens*, these are shown to be related to exocytosis, DNA replication, transcription, and modifications, amino acid activation and transport, RNA related processes, histone modification, and protein modifications (**Table 3**). To cite one example, histone modification functions associated with both organisms include the DNA methyltransferase 1-associated protein (Medtr1g086590) in plants and the KANSL1 (KAT8 Regulatory NSL Complex Subunit 1) histone acetyltransferase in humans.

**Table 3:** Common biological processes shared between *M. truncatula* and *H. sapiens* as resulted from the network-based approach involving the *M. truncatula* network construction. The ID corresponding to each GO term (GO ID) along with putatively target genes and corresponding miRNAs are provided.

Biological process	*M. truncatula*			*H. sapiens*		
	GO ID	Gene	miRNA	GO ID	Gene	miRNA
Exocytosis	GO:0006887	Medtr4g102120	mtr-miR5559-3p	GO:0006887	SNPH	mtr-miR399t-5p
		Medtr8g023330	mtr-miR5558-3p	GO:0006904	RIMS3	mtr-miR482-5p
					SYT1	mtr-miR5211
					SYT2	mtr-miR2640
					NOTCH1	mtr-miR5266
					RAB3GAP1	mtr-miR5209
					RPH3AL	mtr-miR2589
					SYT15	mtr-miR166d
DNA replication, transcription, and modifications	GO:0006261	Medtr4g106540	mtr-miR5741a	GO:0090329	INO80	mtr-miR399t-5p
	GO:0090329			GO:2000104	LIG3	mtr-miR5294a
				GO:0006268	HMGA1	mtr-miR5276 mtr-
				GO:0044030	GRHL2	miR2589
				GO:2000678	PER2	mtr-miR169k
				GO:0032786	SIN3A	mtr-miR156b-3p
					BRD4	mtr-miR5266
Amino acid activation and transport	GO:0043038	Medtr7g083030	mtr-miR2657	GO:0009081	BCKDK	mtr-miR5273
	GO:0043039			GO:0009083	IVD	mtr-miR2640
	GO:0006418			GO:0051955	PER2	mtr-miR169k
				GO:0051957	RAB3GAP1	mtr-miR5209
				GO:0009065	NTSR1	mtr-miR408-3p
					TINAGL1	mtr-miR166d
					NANOS2	mtr-miR160c
					PRODH	mtr-miR169d-3p
RNA related processes	GO:0016071	Medtr3g077320	mtr-miR2629f	GO:0050686	CELF1	mtr-miR2670f
	GO:0006397			GO:0006376	CELF2	mtr-miR399t-5p
	GO:0008380			GO:0061014	GIGYF2	mtr-miR166d
	GO:0000375			GO:0061157	TNRC6B	mtr-miR5211
	GO:0000377			GO:0050686	KHSRP	mtr-miR398b
	GO:0000398				MEX3D	mtr-miR2673a
					RNPS1	mtr-miR398b
					SUPT5H	
Histone modification	GO:0016570	Medtr1g086590	mtr-miR395e	GO:0043981	KANSL1	mtr-miR482-5p
	GO:0016573	Medtr4g108080	mtr-miR156a	GO:0043982		
Protein modifications	GO:0043543	Medtr1g086590	mtr-miR395e	GO:0018345	CLIP3	mtr-miR527
	GO:0006473			GO:0006517	ZDHHC18	mtr-miR168b
	GO:0006475			GO:0036507	MARCH6	mtr-miR390
	GO:0018394			GO:0036508	UGGT1	mtr-miR5270a
	GO:0018393			GO:0042532	NF2	mtr-miR5206b

Taken together, the results obtained confirmed that both network-based approaches lead to consistent conclusions, even if *M. truncatula* is characterized by a less detailed Gene Ontology which prevents strong matching between the two plants.

### Do mtr-miRNAs Putatively Target Genes Involved in DDR in Plants and Humans?

The three bioinformatic approaches used in the present study allowed to search for common biological processes targeted by *M. truncatula* miRNAs in both plant and human cells. Each approach provided different sets of information that can be either complementary or divergent, based on the assumptions of each used methodology. Besides the results presented so far, we also wanted to focus on a particularly conserved pathway in plants and humans, namely DDR (Yoshiyama et al., 2013; Nikitaki et al., 2018), because information relative to miRNAs targeting this essential process is still scarce, especially when concerning plants. Hence, **Tables 4** and **5** summarize a series of processes related to the DDR pathway and downstream processes in both kingdoms.

**Table 4:** Biological processes related to DNA repair, recombination, replication and chromatin remodeling common to *A. thaliana* and *H. sapiens* as resulted from the network-based approach. The ID corresponding to each GO term (GO ID) along with putatively target genes and corresponding miRNAs are provided.

Biological process	*A. thaliana*			*H. sapiens*		
	GO ID	Gene	miRNA	GO ID	Gene	miRNA
DNA repair	GO:0006284	DME	mtr-miR2086-3p	GO:2000779	FOXM1	mtr-miR169d-3p
	GO:0045003	DML1	mtr-miR2651		PPP4C	mtr-miR169k
	GO:0000724	AT1G75230	mtr-miR5240			
		RAD54	mtr-miR172c-5p			
		RECA1	mtr-miR5558-3p			
		ASF1B	mtr-miR1509a-3			
		GMI1	pmtr-miR169l-3p			
		KU80	mtr-miR5272f			
DNA recombination and replication	GO:0006310	ASF1B	mtr-miR1509a-3p	GO:0090329	INO80	mtr-miR399t-5p
		GMI1	mtr-miR169l-3p	GO:2000104	LIG3	mtr-miR5294a
		KU80	mtr-miR5272f	GO:2000678	PER2	mtr-miR169k
		RAD54	mtr-miR172c-5p	GO:0032786	SIN3A	mtr-miR156b-3p
		RCK	mtr-miR5754		BRD4	mtr-miR5266
		RECA1	mtr-miR5558-3p			
		RPA70B	mtr-miR2592a-5p			
Chromatin remodeling	GO:0006306	DME	mtr-miR2086-3p	GO:0043981	KANSL1	mtr-miR482-5p
	GO:0044728	DML1	mtr-miR2651	GO:0043982	HMGA1	mtr-miR5276
	GO:0006305	DRD1	mtr-miR2650	GO:0070828	TNRC18	mtr-miR2589
	GO:0006304	EMB2770	mtr-miR7696c-5p	GO:0031507	GRHL2	mtr-miR2589
	GO:0031056	SDG14	mtr-miR2650	GO:0031936	PHF2	mtr-miR160c
	GO:0031058		mtr-miR2086-3p	GO:0006268	SIN3A	mtr-miR156b-3p
	GO:0031060		mtr-miR7696c-5p	GO:0044030	ZNF304	mtr-miR166e-5p
	GO:0031062			GO:0031935		
	GO:1905269			GO:0031937		
	GO:1902275					
	GO:0080188					

**Table 5:** Biological processes related to cell cycle and cell death common to *A. thaliana* and *H. sapiens* as resulted from the network-based approach. The ID corresponding to each GO term (GO ID) along with putatively target genes and corresponding miRNAs are provided.

Biological process	*A. thaliana*			*H. sapiens*		
	GO ID	Gene	miRNA	GO ID	Gene	miRNA
Cell cycle	GO:0000075	ASF1B	mtr-miR1509a-3p	GO:1901989	BRD4	mtr-miR5266
	GO:0045930	RAD9	mtr-miR2638b	GO:1901992	EIF4G1	mtr-miR166d
	GO:0007093			GO:1902751	PHB2	mtr-miR5266
				GO:0010971	SIN3A	mtr-miR156b-3p
				GO:0071157	MDM2	mtr-miR169k
					MDM4	mtr-miR5266
Cellular senescence	GO:0000723	KU80	mtr-miR5272f	GO:2000772	ABL1	mtr-miR5276
		TRB1	mtr-miR5558-5p		HMGA1	mtr-miR5276
					VASH1	mtr-miR160c
Apoptosis/cell death	–	–	–	GO:1902108	BMF	mtr-miR2613
				GO:1902110		mtr-miR5266
				GO:1902263	GDNF	mtr-miR2673a
				GO:0060561	BCL2L11	mtr-miR5273
				GO:0001844		mtr-miR5266
				GO:1900117	NOTCH1	mtr-miR5266
				GO:0070231	VDR	mtr-miR5276
				GO:0043525	YWHAG	mtr-miR2673a
				GO:1901028		mtr-miR399t-5p
				GO:1901216	DFFA	mtr-miR399t-5p
				GO:1901030	TP53BP2	mtr-miR399t-5p
				GO:2001238		mtr-miR5266
				GO:0097192		mtr-miR2613
				GO:1900740		mtr-miR160c
				GO:1902686	AKT1	mtr-miR160c
						mtr-miR5266
					DFFA	mtr-miR399t-5p
					KDELR1	mtr-miR166d
					ARHGEF7	mtr-miR2589
					GDNF	mtr-miR2673a
					BAD	mtr-miR5266
						mtr-miR5276
						mtr-miR399t-5p
						mtr-miR2673a
					ABL1	mtr-miR399t-5p
					ITM2C	mtr-miR5206a
					PEA15	mtr-miR160c
					TNFRSF12A	mtr-miR160c
					TRAF2	mtr-miR2673a
					CX3CL1	mtr-miR2613
					GDNF	mtr-miR5211
					SPG7	mtr-miR399t-5p

DNA repair, recombination, replication, and chromatin dynamics are tightly connected, as modifications of DNA conformation is required in order to allow access of the repair machinery to the damaged sites. This interplay is evidenced also by the fact that several genes are shared among these processes; for instance, the *A. thaliana* DME (Demeter) and DML1 (Demeter-like 1) are associated with both DNA repair (BER-base excision repair, GO:0006284) and chromatin modification-related functions (GO:0006306, GO:0044728), whereas RAD54 (DNA Repair and Recombination Protein), RECA1 (Recombination A1 protein), and KU80 (helicase Ku80 subunit of KU complex) are coupled with DNA repair (DSB, double-strand break repair, GO:0045003; HR-homologous recombination, GO:0000724) and DNA recombination (GO:0006310) processes (**Table 4**). Similarly, literature available from medical research assigned roles in DNA damage repair and chromatin remodeling to some of the genes predicted as targets of mtr-miRNAs. To cite some examples, PPP4C (Protein Phosphatase 4 Catalytic Subunit), is involved in a myriad of processes spanning from microtubule organization, to apoptosis, DNA repair, DNA damage checkpoint signaling, regulation of histone acetylation (Zhou et al., 2002; Lee et al., 2010), while INO80 (INO80 Complex Subunit) is the catalytic ATPase subunit of the INO80 chromatin remodeling complex, being however related also to DNA DSB repair (Conaway and Conaway, 2009). Functions related to DNA and chromatin/histone modifications were identified also in the *M. truncatula* network-based approach (see **Table 3**). This is the case of Medtr1g086590 (DNA methyltransferase 1-associated protein), Medtr4g108080 (ubiquitin-conjugating enzyme), and Medtr4g106540 (E2F transcription factor-E2FE-like protein) accessions. Within the alignment-based approach, mtr-miR2589 was predicted to target the *M. truncatula* Medtr6g047800 (tRNA methyltransferase complex GCD14 subunit) and the *H. sapiens* SETD1A (SET Domain Containing 1A, Histone Lysine Methyltransferase), functions involved in chromatin organization in both organisms (**Supplementary Table 2**).

Other processes tightly correlated with DDR include cell cycle and cell death (apoptosis/necrosis/programmed cell death). DNA replication, recombination, and repair are more active during certain phases of the cell cycle and the success of these processes can decide the fate of the cell. The connection between pathways is evidenced by genes that play important functions in both DNA repair/replication, chromatin remodeling, and cell cycle/cell death (**Table 5**). This is the case of the ASF1B (Anti-Silencing Function 1B, histone chaperone) and KU80 functions in plants, involved in the S‐phase replication‐dependent chromatin assembly (Zhu et al., 2011) and maintenance of genome integrity (West et al., 2002), respectively, and the SIN3A (Histone Deacetylase Complex Subunit Sin3a) and HMGA1 (High Mobility Group Protein A1) genes in humans, with roles associated to chromatin regulation and cell cycle progression (Silverstein and Ekwall, 2004; Pierantoni et al., 2015). While the *A. thaliana* network-based approach has not identified genes (predicted targets of mtr-miRNAs) associated with cell death in plants, this however led to the identification of many putative targets related to apoptosis in human cells. To cite a few, BCL2L11 (BCL2-Like 11 apoptosis facilitator), NOTCH1 (Notch Receptor 1), and TP53BP2 (Tumor Protein P53 Binding Protein 2) are among the most essential apoptotic factors. NOTCH1, part of the Notch signaling pathway, is involved in many processes related to cell fate specification, differentiation, proliferation, and survival, while its activation is related to many types of cancers (e.g. cervical, colon, head and neck, lung, renal, pancreatic, leukemia, and breast cancer) (Xiao et al., 2016). Hence, dietary miRNAs targeting this specific function may have positive implications in sustaining cancer therapies. The alignment-based approach allowed to identify a conserved miRNA (mtr-miR319d-5p) predicted to target genes associated with cell death functions in both *M. truncatula* (DCD-development and cell death domain protein) and *H. sapiens* (MESD, PRR5L) (**Supplementary Table 2**). While MESD (Mesoderm Development LRP Chaperone) is related to the Notch pathway (Hsieh et al., 2003), PRR5L (Proline Rich 5 Like) regulates the activity of the mTORC2 (mechanistic target of rapamycin) complex controlling cell migration (Gan et al., 2012).

Hence, to answer the herein proposed question, the network-based as well as the alignment-based approaches pinpointed mtr-miRNAs predicted to target genes involved in DDR and downstream processes in *A. thaliana*, *M. truncatula*, and *H. sapiens*.

## Discussion

**Figure 5:**
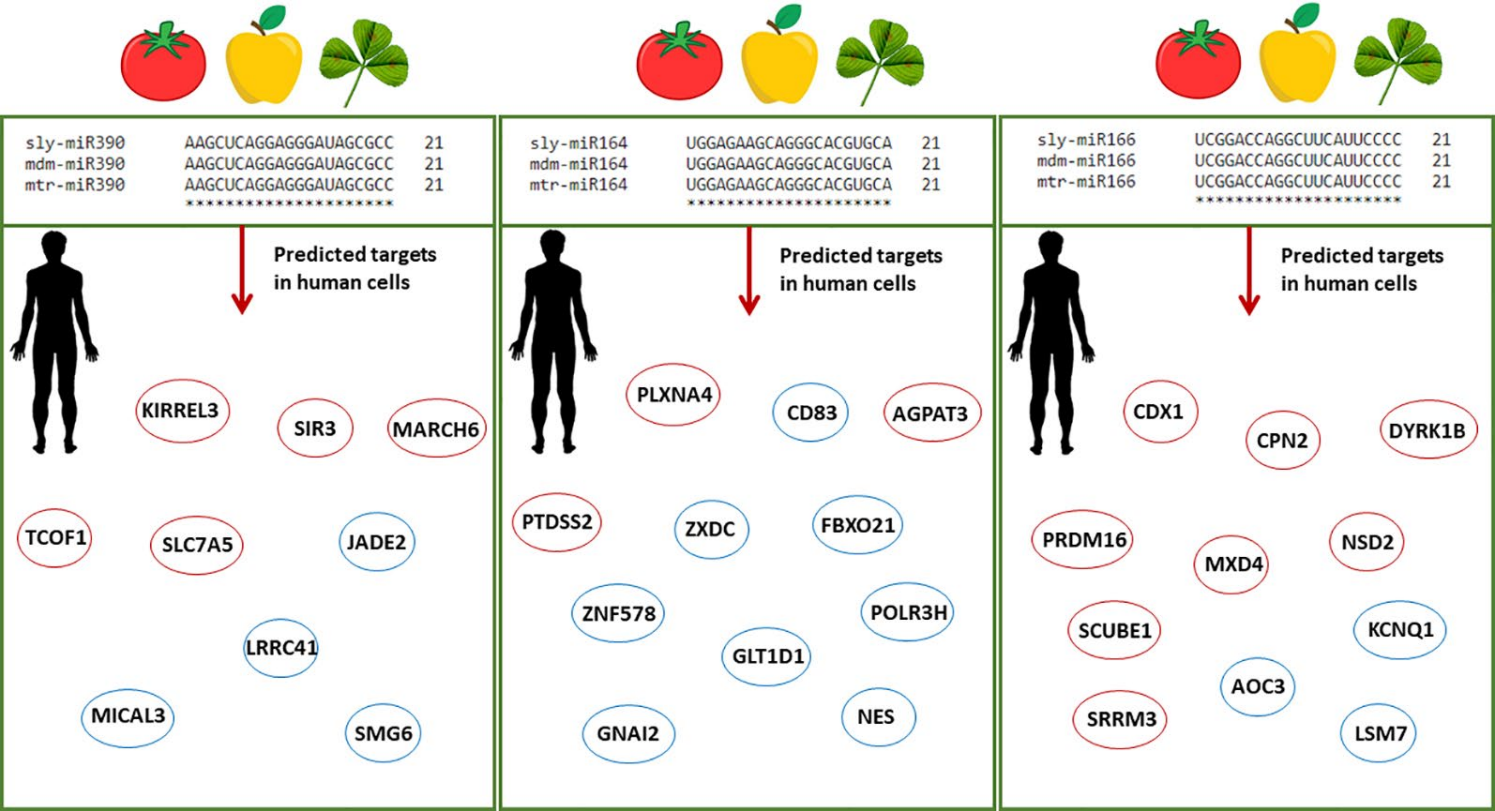
Schematic representation of conserved plant miRNAs potentially targeting human genes. Alignments between three conserved miRNAs (miR390, miR164, miR166) from different plant species, namely *Solanum lycopersicum* (sly), *Malus domestica* (mdm), and *M. truncatula* (mtr), show 100% sequence similarity. The predicted human target genes found in the enriched biological processes of the network-based approach and among the genes with significant sequence similarity in the alignment-based approach are shown in red and blue circles, respectively. Abbreviations: KIRREL3, Kirre Like Nephrin Family Adhesion Molecule 3; SIRT3, Sirtuin 3; MARCH6, Membrane Associated Ring-CH-Type Finger 6; TCOF1, Treacle Ribosome Biogenesis Factor 1; SLC7A5, Solute Carrier Family 7 (Amino Acid Transporter Light Chain, L System), Member 5; JADE2, Jade Family PHD Finger 2; LRRC41, Leucine Rich Repeat Containing 41; MICAL3, Microtubule Associated Monooxygenase, Calponin And LIM Domain Containing 3; SMG6, Nonsense Mediated MRNA Decay Factor; PLXNA4, Plexin A4; AGPAT5, 1-Acylglycerol-3-Phosphate O-Acyltransferase 5; PTDSS2, Phosphatidylserine Synthase 2; CD83, Cluster of Differentiation 83; ZXDC, ZXD Family Zinc Finger C; FBXO21, F-Box Protein 21; GLT1D1, Glycosyltransferase 1 Domain Containing 1; GNAI2, G Protein Subunit Alpha I2; ZNF578, Zinc Finger Protein 578; NES, Nestin; POLR3H, RNA Polymerase III Subunit H; CDX1, Caudal Type Homeobox 1; CPN2, Carboxypeptidase N Subunit 2; DIRK1B, Dual specificity tyrosine-phosphorylation-regulated kinase 1B; MXD4, MAX Dimerization Protein 4; NSD2, Nuclear Receptor Binding SET Domain Protein 2; PRDM16, Histone-lysine N-methyltransferase PR/SET Domain 16; SCUBE1, Signal Peptide, CUB Domain And EGF Like Domain Containing 1; SRRM3, Serine/Arginine Repetitive Matrix 3; AOC3, Amine Oxidase Copper Containing 3; KCNQ1, Potassium Voltage-Gated Channel Subfamily Q Member 1; LSM7, U6 snRNA-associated Sm-like protein 7.

In view of the controversies raised by the recent ‘dietary xenomiR’ hypothesis (Witwer, 2012), bioinformatics studies have the potential to aid the ongoing efforts to reinforce new methodologies and provide the basis for further experimental validation. Model organisms, like *A. thaliana*, are used as guidance systems to explore bioinformatics data-driven questions related to putative cross-species miRNA targets (Zhang et al., 2016a). The high interest in this field prompted the development of databases able to predict the functional impact of food-borne miRNAs in humans (Chiang et al., 2015; Shu et al., 2015). The DMD (Dietary MicroRNA Database) database covers only very few edible plant species (Chiang et al., 2015), hence there is the need to substantially enlarge the information and include alternative species with a potential impact on food security. Within this framework, the current study aims at identifying plant miRNAs along with their endogenous and cross-kingdom targets to pinpoint conserved pathways between evolutionary distant species. Starting from a list of publicly available *M. truncatula* miRNAs, we made the assumption that any miRNA may have the potential to target genes in both plants and humans. Given that the bioinformatics approaches do not allow the prediction of miRNAs stability and function validation within the organisms, there is the need to further experimentally confirm the proposed hypotheses. The choice of plant species is driven by the fact that *M. truncatula* is at the crossroad between model organisms (in the case of legume research) and economically relevant species, given its potential use as microgreens to support more sustainable agriculture. The presented methodologies can serve both as guidelines to be applied to other plant species as well as to test new hypotheses exploring the potential benefits of food-borne mtr-miRNAs targeting human genes. When considering the conserved families of miRNAs, this study could aid the translational research covering other economically relevant plant species (with 100% sequence similarity) and potential human target genes. As exemplified in **Figure 5**, miR164, miR166, and miR390 have a 100% sequence similarity between *M. truncatula* and other dicot plant species such as tomato (*Solanum lycopersicum*) and apple (*Malus domestica*). Among the selected examples, miR166 was previously demonstrated to be abundant in different human body fluids and tissues (Lukasik et al., 2018; Zhao et al., 2018b). The putative human targets identified either through the network- or alignment-based approaches could serve as potential candidates to aid medical interventions. To cite one example, inhibition of the AOC3 (Amine Oxidase Copper Containing 3), playing important roles in adipogenesis and putatively targeted by miR166, could result in decreased fat deposition (Carpéné et al., 2007; Shen et al., 2012), hence addressing the big issues related to obesity and the many obesity-associated diseases.

It is important to underline that the experimental design of this study was thought in such a way to potentiate the identification of conserved pathways/players between evolutionary distant species. To do so, three different bioinformatics pipelines were developed (two network-based approaches, considering *A. thaliana* and *M. truncatula* model species, and one alignment-based approach) to confront plant and human targeted biological processes. Form a methodological point of view, the developed approaches enable the exploration of different assumptions supported by robust statistical methods. The network-based approaches rely on extensive knowledge available on the interactions among miRNA target genes in a given species. The knowledge was exploited for the construction of gene co-expression/interaction networks, from which a set of biological processes predicted to be targeted by the plant miRNAs were found. This approach aims to study the regulatory potential of the full list of *M. truncatula* miRNAs. Two network-based approaches were implemented, differing from the point of view of plant network construction. In one approach, the predicted *M. truncatula* target genes were mapped to the genome of *A. thaliana*, which is a supported organism in widely used network construction tools, while *M. truncatula* is not. The other network-based approach relies on the construction of a novel co-expression network for *M. truncatula*, using publicly available expression data, thereby evaluating the robustness of the performed assumptions on the plant network. Finally, the alignment-based approach was radically different, since it only relies on target gene and protein sequences, with no other assumption, and aimed to discover sequence similarities between plant and human targets, individually for each targeting miRNA. In this context, this approach enabled the inference on the potential effect of every single miRNA of the initial list. Importantly, none of the proposed strategies is focused on the prediction of individual target genes: only the ones sharing statistically over-represented processes (in network-based approaches), or having the same targeting miRNA and a statistically significant nucleotide and protein sequence similarity (in alignment-based approaches) were detected and discussed in this work. The prediction of individual targets relied on existing computational tools used in the early steps of the workflow under assumptions similar to the ones of previous studies (Zhang et al., 2016a), despite no standardized pipeline is well-accepted to accomplish this task due to the lack of genome-wide experimental validations across species. The main limitations of the proposed approaches are that: *(i)* no assumption was made on which miRNAs can be delivered between plants and human, *(ii)* only the 3′ UTR region was assumed to be the target region of plant miRNAs in humans. In addition, we decided to rely on computational methods to predict the binding between miRNA and putative target transcript; alternative approaches could also exploit homology between plant and human miRNAs, which might share the same seed region and then identify human targets based on experimentally validated target genes in human cells (Liu et al., 2017). However, such alternative would have led to the consideration of a smaller number of plant miRNAs, since only the ones with homologous features could have been included in the analysis, thereby losing the possibility to study the whole plant miRNA regulation potential. Nonetheless, we believe that the methodological approaches are sufficiently general to be extended onto the desired miRNA list and candidate target list (e.g. 3′/5′ UTRome, transcriptome, or collection of coding sequences) as input. Moreover, the interpretation of the enriched biological processes identified from network analysis is affected by Gene Ontology terms of different specificity and name (Zhang et al., 2016a), thereby limiting the discovery of all the potentially targeted functions.

From a biological perspective, the employed strategies resulted in both complementary and divergent observations. For instance, ‘exocytosis’ was a common denominator in all three investigated species (*M. truncatula*, *A. thaliana*, and *H. sapiens*) when using the network-based approaches. On the other hand, the alignment-based approach allowed a more direct identification of miRNAs targeting genes in *M. truncatula* and *H. sapiens* (e.g., same miRNA *vs.* similar/different functions) whereas the generated networks illustrates conserved biological processes (e.g., same function *vs.* same/different miRNAs). The two approaches also indicated connecting elements. For example, mtr-miR168a was predicted to target AGO1 in plants and PVP in humans, functions associated with pathogen (namely, viruses) defence, in both approaches. The miR168a is part of a conserved family of plant miRNAs among different species, but as we seen in **Supplementary Figure 2** and other cited literature (Lang et al., 2019), differences exist between dicot and monocot species. The predicted human targets observed in previously published researches (Zhang et al., 2012; Javed et al., 2017), were not found in the enriched process or sequence similarity with our approaches (see **Supplementary Table 1**). This can have different explanations: *(i)* four sequence mismatches (two located within the seed region) are present between osa-miR168a and mtr-miR168a, including a G at position 14, recently reported to generate a G:U wobble that limits its binding to LDLRAP1 (Lang et al., 2019); *(ii)* only 3′ UTR regions were considered in our study, and since osa-miR168a targets the LDLRAP1 CDS region (Zhang et al., 2012), we did not find this match in the target list; *(iii)* we used the entire length of the miRNA and 100% sequence complementarity instead of only the miRNA seed region (Zhang et al., 2012; Javed et al., 2017). By searching for mtr-miR168a and osa-miR168a targets in the full transcript sequences, a more relevant annealing score to the LDLRAP1 gene was found with osa-miR168a than mtr-miR168a, probably due to the sequence mismatches.

Considering that the purpose of the study was to identify conserved functions between distant species through the lens of mtr-miRNAs, our results report ‘vesicle docking involved in exocytosis’, ‘modulation by virus of host morphology or physiology’, ‘cellular response to virus’, ‘positive regulation of posttranscriptional gene silencing’, and ‘branched-chain amino acid metabolic process’ as common biological processes between Arabidopsis and humans (**Table 1**). A different study designed to look into the role of plant miRNAs in inter-species regulatory networks indicated ion transport and stress response as shared functions between Arabidopsis and humans (Zhang et al., 2016a). However, this study took into consideration only 25 miRNAs to construct the relative species-specific networks while we started from a list of 426 *M. truncatula* miRNAs to disclose the full regulatory potential. When considering the alignment-based approach, the most represented predictive targets in *M. truncatula* covered transcription factors and hormone-responsive genes. Interestingly, some of these miRNAs (e.g., mtr-miR160a, mtr-miR164b, mtr-miR166d, mtr-miR2673a) were predicted to target TFs (HAND2, ZXDC) and hormone-related functions (DYRK1B, HNF4A) also in human cells. This is in agreement with the concept that miRNAs may behave in a hormone-like manner since hormones and miRNAs are reciprocally regulated in both plant and animal kingdoms (Li et al., 2018).

Because evidence of miRNAs involvement in the regulation of DDR-related pathways is still limited in plants and considering the conservation of some DDR functions between plants and animals (Yoshiyama et al., 2013; Nikitaki et al., 2018), we decided to focus our attention on these specific pathways. Hence, miRNAs predicted to target genes involved in DNA repair, recombination, and replication, chromatin remodeling, cell cycle, and cell death were hereby identified in plants (see **Tables 4** and **5**). For instance, mtr-miR172c-5p, mtr-miR2638b, mtr-miR5272f, and mtr-miR2086-3p, were predicted to target the Arabidopsis RAD54, RAD9, KU80, and DME genes, respectively. In the *M. truncatula* network-based approach, the ‘DNA-dependent DNA replication’ (GO:0006261) biological process is represented by Medtr4g106540 (E2F transcription factor-E2FE-like protein) as a predicted target of mtr-miR5741a (see **Supplementary Dataset 3**). Within the alignment-based approach, mtr-miR2589, mtr-miR482-5p, mtr-miR5287b, and mtr-miR319d-5p were predicted to target two methyltransferases (Medtr6g047800, Medtr5g079860), the CDC48 (Medtr7g088980), and DCD genes (Medtr4g084080) (see **Table 2** and **Supplementary Table 2**). All these hits bring an added value for plant science as they associate specific, previously unknown, miRNAs to the regulation of DDR functions. To date, there are only a few reports predicting DDR-associated functions as putative targets of miRNAs; for instance, MRE11 (Meiotic Recombination 11, a DSB repair nuclease) has been predicted as target of miR5261 in *Citrus sinensis* (Liang et al., 2017), or XPB2 (*Xeroderma pigmentosum* type B, an excision repair helicase) predicted as target of miR122c-3p in *Triticum aestivum* (Sun et al., 2018). In human cell research, miRNAs involvement in the regulation of DDR is much more advanced and it is associated with the development of new therapeutic/diagnostic tools (Hu and Gatti, 2011; He et al., 2016). A number of studies document that p53, the master-regulator of DDR, is targeted by endogenous miRNAs (Hu et al., 2010; Kumar et al., 2011). This is the case of miR-25 and miR-30d, associated with p53 downregulation along with the suppression of downstream interactors p21, BAX, and Puma, hence being involved in apoptotic processes (Kumar et al., 2011). Another example, in relation to DNA repair pathways, indicates that hsa-miR-526b targets the Ku80 mRNA, with subsequent alterations of DSB repair and cell cycle arrest (Zhang et al., 2015). Our bioinformatics approach also revealed mtr-miRNAs predicted to target human genes with roles in DNA repair and related processes (see **Table 4**). To reiterate some examples, PPP4C (putatively targeted by mtr-miR169d-3p) catalyses the dephosphorylation of RPA2 (Replication Protein A2) in response to DNA damage, thus permitting the recruitment of RAD51 (an essential recombinase for the HR repair) to the damaged site (Lee et al., 2010). Likewise, FOXM1 (Forkhead Box M1), predicted as a target of mtr-miR169k, is among the most overexpressed oncoproteins in many types of cancer and therapeutic interventions to suppress its function are of great interest (Halasi et al., 2018). Hence, the identification of a miRNA belonging to conserved plant miRNA families (in this case, miR169) as a putative target of this gene may bring further support to ongoing cancer remedies. In relation to this, also many of the predicted targets associated with apoptosis (see **Table 5**), like the presented example of NOTCH1 (putatively targeted by mtr-miR5266), could have similar implications. The use of plant miRNAs as adjuvants in cancer therapies has been already tested; for example, plant miR159, abundantly found in human serum, has been associated with reduced incidence and progression of breast cancer because it targets the TCF7 (a Wnt signaling transcription factor) gene, causing decreased levels of MYC (Avian Myelocytomatosis Viral Oncogene Homolog) proteins, essential for cell cycle progression (Chin et al., 2016).

In conclusion, the current study provides comprehensive datasets (obtained by combining *ad-hoc* bioinformatics tools) related to *M. truncatula* miRNAs potential to putatively target genes across evolutionary distant species. By focusing on specific DDR-related functions, the hereby presented results significantly contribute to enriching the current knowledge regarding the conservation of DDR in plant and human cells. Considering the implications that some of these putative interactions could have for the biomedical sector, this study also offers additional hypotheses to be further experimentally validated. The developed pipeline can be applied to any species of interest to address species-specific cross-kingdom interactions or to carry out large-scale investigations involving a number of plant/animal species. The application of the proposed methods to other case-studies should take into account the following considerations on data, software, and knowledge availability: *(i)* the miRNAs of a ‘donor’ organism (e.g., a plant) and the transcriptome of the ‘donor’ and ‘receiving’ (e.g., an animal) organism should be available from public datasets or *de-novo* sequencing, annotation and expression studies; *(ii)* the miRNA dataset could be further refined by selecting experimentally known or computationally predicted miRNAs that are protected from degradation during incorporation in the receiver organism. The prediction miRNA targets in both species can be carried out *via* bioinformatic tools online available, although tool specificity for the target species should be taken into account and the parameter(s) of the algorithms should be fine-tuned accordingly, in order to have a balanced number of targets to be analysed in both species. Other target prediction algorithms may be used to overcome the so far weak knowledge on cross-kingdom regulation mechanisms to identify the target transcripts of heterologous miRNAs. For the miRNA target network reconstruction, an homology-based strategy should exploit the homology between the desired organisms and their model organisms in the same kingdom (e.g., *A. thaliana* for plants); on the other hand, a *de-novo* organism-specific co-expression network reconstruction relies on the availability of gene expression data from public datasets (e.g., GEO) or novel microarray/RNAseq experiments. All the networks can be analysed *via* specific software (e.g., Cytoscape) to find clusters of co-expressed genes and to carry out enrichment analyses on the desired gene sets. The experimental validation of the predicted targets can be subsequently performed *via* degradome analysis.

## Data Availability Statement

All datasets generated for this study are included in the article/**Supplementary Material**.

## Author Contributions

MB, AM, and LP conceptualized the study. AM and LP wrote the manuscript. DM performed the network-based approach, MB performed the alignment-based approach, and ES performed the *M. truncatula* network reconstruction and analysis. AM, MB, and CG analysed the generated data. All authors read and approved the manuscript.

## Funding

Authors acknowledge the funding received from FFABR-ANVUR (Funding for Basic Activities Related to Research—Italian National Agency for the Evaluation of Universities and Research Institutes), the University of Pavia Crowdfunding (UNIVERSITIAMO) campaign “The other side of the seed” (https://universitiamo.eu/en/campaigns/the-other-side-of-the-seed/), and the Italian Ministry of Education, University and Research (MIUR): *Dipartimenti di Eccellenza* Program (2018–2022)—Dept. of Biology and Biotechnology “L. Spallanzani”, University of Pavia.

## Conflict of Interest

The authors declare that the research was conducted in the absence of any commercial or financial relationships that could be construed as a potential conflict of interest.
