# Supplementary material for: A Bioinformatics Approach to Explore MicroRNAs as Tools to Bridge Pathways Between Plants and Animals. Is DNA Damage Response (DDR) a Potential Target Process?
Source: Front Plant Sci. 2019 Nov 26;10:1535. doi: 10.3389/fpls.2019.01535 (PMC6901925; doi:10.3389/fpls.2019.01535)
Supplement: Supplementary file 1 [file Table_1.docx]

Supplementary Material

**A bioinformatics approach to explore microRNAs as tools to bridge pathways between plants and animals: a focus on DNA Damage Response**

**Massimo Bellato^1^, Davide De Marchi^1^, Carla Gualtieri^2^, Elisabetta Sauta^1^, Paolo Magni^1^, Anca Macovei^2,*^, Lorenzo Pasotti^1,*^**

^1^Laboratory of Bioinformatics, Mathematical Modelling and Synthetic Biology, Department of Electrical, Computer and Biomedical Engineering - Centre for Health Technology, University of Pavia, Italy

^2^Plant Biotechnology Laboratory, Department of Biology and Biotechnology “L. Spallanzani”, University of Pavia, Italy

*** Correspondence:**A. Macovei, [anca.macovei@unipv.it](mailto:anca.macovei@unipv.it)

L. Pasotti, [lorenzo.pasotti@unipv.it](mailto:lorenzo.pasotti@unipv.it)


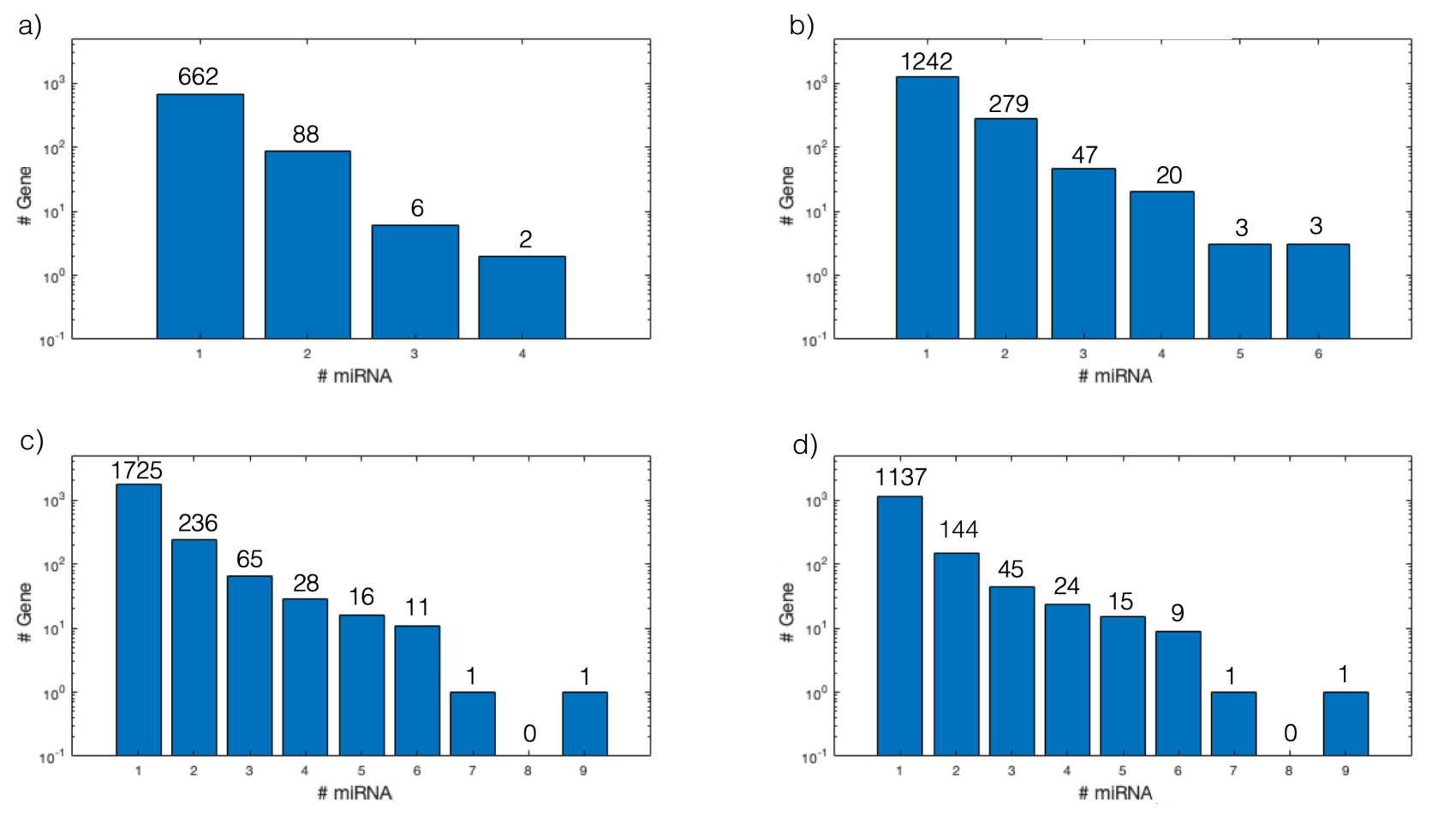


**Supplementary Figure 1.** Count of the *Homo sapiens* and *Medicago truncatula* miRNA targets in the two bioinformatics pipelines. Bars represent the numbers of predicted genes that are targeted by one or more miRNAs. **(a)** Gene targets derived from *H. sapiens* network-based pipeline. **(b)** Gene targets derived from the *H. sapiens* alignment-based pipeline. **(c)** Gene targets derived from the *M. truncatula* network-based pipeline. **(d)** Gene targets derived from the *M. truncatula* alignment-based pipeline. The y-axis is shown in logarithmic scale to better visualize the bars with low number of target genes.

**(a)**

osa-miR168a U**C**GCUUGGUGC**A**G**A**UCGGGA**C** 21

mtr-miR168a U**U**GCUUGGUGC**U**G**G**UCGGGA**A** 21

* ********* * ******

**(b)**


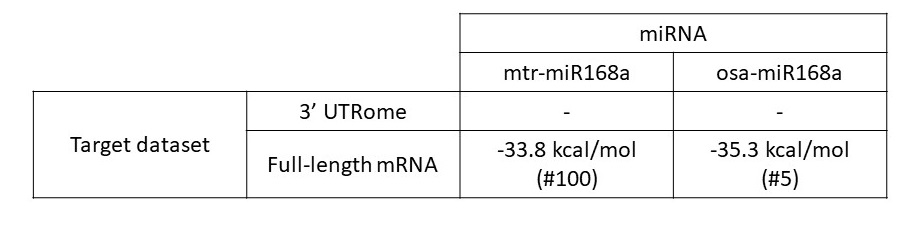


**Supplementary Figure 2**. **(a)** Sequence alignment between osa-miR168a (*O. sativa*) and mtr-miR168a (*M. truncatula*) performed using the Clustal Omega online tool (<https://www.ebi.ac.uk/Tools/msa/clustalo/>). **(b)** Summary of LDLRAP1 target prediction results using mtr-miR168a or osa-miR168a, and 3’ UTRome or full-length transcripts as dataset. Numbers outside brackets represent the best MFE score (kcal/mol), and numbers in brackets indicate the position of LDLRAP1 among the target list ranked by MFE.’-’, no targets found.

**Supplementary Table 1**. Predicted genes targeted by mtr-miR168 in human cells as revealed by the network-based and alignment-based approaches.

| Network-based approach | | | Alignment-based approach | |
| --- | --- | --- | --- | --- |
| Predicted target | **Gene description** | **GO Term** | **Predicted target** | **Gene description** |
| ST8SIA1 | Sialyltransferase 8A | sphingolipid biosynthetic process | CDAN1 | Codanin 1 |
| RGS6 | Regulator of G Protein Signaling 6 | regulation of G-protein coupled receptor protein signaling pathway | NISCH | Nischarin |
| IL18RAP | Interleukin 18 Receptor Accessory Protein | positive regulation of natural killer cell mediated immunity | CEMP1 | Cementum Protein 1 |
| PVR | Poliovirus Receptor | positive regulation of natural killer cell mediated immunity | PVR | Poliovirus Receptor |
| SYN2 | Synapsin 2 | neurotransmitter secretion | HTRA3 | HtrA Serine Peptidase 3 |
| PPFIA1 | Protein Tyrosine Phosphatase Receptor Type F Polypeptide-Interacting Protein Alpha-1 | regulation of actin filament bundle assembly | ZNF710 | Zinc Finger Protein 710 |
| ZDHHC18 | Zinc Finger DHHC Domain-Containing Protein 18 | protein palmitoylation |  |  |
| B3GAT1 | Beta-1,3-Glucuronyltransferase 1 | glycosaminoglycan biosynthetic process |  |  |

**Supplementary Table 2**. Examples of mtr-miRNAs and their putative target genes in *M. truncatula* and *H. sapiens* as revealed by the alignment-based approach. The genes and their respective accessions are provided for each organism.

| mtr-miRNA | *M. truncatula* | | *H. sapiens* | |
| --- | --- | --- | --- | --- |
|  | **Accession** | **Gene** | **Accession** | **Gene** |
| mtr-miR2600e | Medtr2g089765 | anthocyanin 5-aromatic acyltransferase | NM_020894 | UVSSA |
| mtr-miR5285b | Medtr8g105290 | nuclear pore complex Nup155-like protein | NM_000370 | TTPA |
| mtr-miR319d-5p | Medtr4g134770 | translation elongation factor EF1B, gamma chain | NM_015154 | MESD |
|  | Medtr4g084080 | DCD (development and cell death) domain protein | NM_001160169 | PRR5L |
| mtr-miR2589 | Medtr1g103100 | 40S ribosomal protein S3a-1 | NM_003565 | ULK1 |
|  | Medtr6g047800 | tRNA methyltransferase complex GCD14 subunit | NM_014712 | SETD1A |
| mtr-miR482-5p | Medtr5g079860 | 23S rRNA m2A2503 methyltransferase | NM_014747 | RIMS3 |
|  |  |  | NM_012318 | LETM1 |
| mtr-miR5286b | Medtr4g038400 | ribosomal protein S12/S23 family protein | NM_001010858 | RNF187 |

**Supplementary Table 3.** Common biological processes identified in *A. thaliana* and *M. truncatula* by comparing their corresponding constructed networks. The ID corresponding to each GO term (GO ID) along with putatively target genes/accessions and corresponding miRNAs are listed.

| Biological process | GO ID | *A. thaliana* | | *M. truncatula* | |
| --- | --- | --- | --- | --- | --- |
|  |  | **Gene** | **miRNA** | **Gene** | **miRNA** |
| Exocytosis | GO:0006887 | DAW1  EXO70B1  EXO70D1  EXO70H7  KEU  SEC5A  SEC8  SEC10 | mtr-miR2587e  mtr-miR5253  mtr-miR5244  mtr-miR2653a  mtr-miR397-5p  mtr-miR5559-3p  mtr-miR7698-5p  mtr-miR2679a  mtr-miR5558-3p | Medtr4g102120  Medtr8g023330 | mtr-miR5559-3p  mtr-miR5558-3p |
| Folic acid-containing compound metabolic/ biosynthetic process | GO:0006760  GO:0009396 | AT3G07270  DFB | mtr-miR2606c  mtr-miR2662 | Medtr3g069160 | mtr-miR2662 |
| Thylakoid membrane organization | GO:0010027 | APG3  SCY2 | mtr-miR5212-3p  mtr-miR2590j | Medtr7g108300 | mtr-miR5212-3p |

**Supplementary Datasets are provided as separate excel files.**

**Supplementary Dataset 1.** Network-based analysis datasheet relative to *Homo sapiens* and *Arabidopsis thaliana*. For each species, the genes of each cluster are reported, using gLay and ClusterOne as clustering algorithms. Enriched Biological Processes are reported for each cluster (GO Terms column), as well as the genes relative to each term and the miRNAs (in brackets) targeting each gene (Genes and associated miRNAs column). The "GeneMania" label refers to the fact that the gene is not in the list of miRNA targets but it has been included by GeneMania as interactor for the target network construction.

**Supplementary Dataset 2.** Datasheet containing the results of CDS and protein sequences alignments between *H. sapens* and *M. truncatula* transcript targets. Abbreviations: miRNA, miRNA code; MTGD_ID, *M. truncatula* gene code; NM, *H. sapiens* NCBI RefSeq transcript code from which CDS was retrieved; Name, gene name; CDS, DNA coding sequence; AA, protein sequence; p-val CDS, p-value of alignment between human and plant CDS; p-val CDS AA, p-value of alignment between human and plant protein sequence.

**Supplementary Dataset 3.** List of *M. truncatula* Biological Process GO terms enriched considering all the found clusters (GO Terms column). Genes relative to each term and the miRNAs (in brackets) targeting each gene (Genes and associated miRNAs column). The "interactor" label refers to the fact that the gene is not in the list of miRNA targets but it has been included by the network reconstruction algorithm as interactor.
